# Perception of Patient Safety Culture in the Framework of the Psychosocial Care Network in Western Amazon: A Cross-Sectional Study

**DOI:** 10.3390/healthcare8030289

**Published:** 2020-08-23

**Authors:** Marcos Cordeiro Araripe, Glauco Martins Silva, Marcos Venicius Malveira de Lima, Ítalla Maria Pinheiro Bezerra, Walédya Araújo Lopes de Melo, Gabriel Zorello Laporta

**Affiliations:** 1 Pós-Graduação em Ciências da Saúde do Centro Universitário Saúde ABC (FMABC), Santo André 9060-870, Brazil; glaucoczs@hotmail.com (G.M.S.); marcos.malveira@ac.gov.br (M.V.M.d.L.); gabriel.laporta@fmabc.br (G.Z.L.); 2 Secretaria de Estado de Saúde do Acre (SESACRE), Rio Branco 69900-333, Brazil; waledya.melo@hotmail.com; 3 Laboratório de Delineamento de Estudos e Escrita Científica do Centro Universitário Saúde ABC (FMABC), Santo André 09060-870, Brazil; italla.bezerra@emescam.br; 4 Universidade Federal do Acre (UFAC) Campus Floresta em Cruzeiro do Sul, Rio Branco 69895-000, Brazil; 5 Escola Superior de Ciências da Santa Casa de Misericórdia (EMESCAM), Vitória 29027-502, Brazil; 6 Curso de Medicina do Centro Universitário UNINORTE, Rio Branco 69915-901, Brazil

**Keywords:** Amazon basin, patient safety culture, HSOPSC, psychosocial care network

## Abstract

The culture of patient safety should be considered a guiding principle for different areas of health. This research presents the results of an analysis on Patient Safety Culture (PSC), according to the perception of health professionals who work in the Psychosocial Care Network, through a descriptive observational cross-sectional study, using the Hospital Survey on Patient Safety Culture in a municipality in the Western Amazon of Brazil. Sixty-nine (69) professionals expressed that the best dimensions evaluated were: “expectations and actions to promote the safety of supervisors and managers” (75%) and “support from hospital management to patient safety” (64%). The worst evaluations were: “non-punitive responses to errors” (27%) and “general perceptions about patient safety” (35%), demonstrating that there still is a culture of fear of causing harm and the need for educational actions on patient safety. In general, all professionals have close contact with patients, regardless of the length on duty; however, the weekly workload and turnover in this sector is leading to a greater chance of errors. The analysis of the internal reliability of the dimensions ranged from 0.12 to 0.89. Only one-third of the respondents scored PSC as “Good” in the studied institutions and 63 out 69 professionals did not report any adverse events in the last 12 months. There are weaknesses in the observed perception of PSC and the obtained results show opportunities and challenges for improvements in the study system.

## 1. Introduction

Patient Safety Culture (PSC) should be considered a guiding principle for different health areas. In medicine, this principle derives from the maxim of medical ethics “primum non nocere”, also called the principle of non-maleficence, which proposes the obligation not to infringe intentional harm. The theme is so important that the World Health Organization (WHO) conceptualized “patient safety” as the absence of real or potential harm, related to health services, transforming this item into a quality indicator, guiding good clinical practices, through safer strategic measures to prevent harm to the patient [1,2]. In the last decade, there has been growing interest from researchers and health professionals in patient safety [3,4]. Thus, the level of commitment of an organization’s professionals to the continuous promotion of a safe therapeutic environment and the influence on safety behaviors and results, for both health professionals and patients, were highlighted [5,6].

By constituting itself as a theme whose notoriety has been increasing in visibility in several countries, including Brazil, the understanding of the environment and the context in which it is located influences changes in behavior, attitude, and organization of the system [5]. In addition, such practices contribute significantly to the organization of a positive safety culture, focusing on communication based on mutual cooperation, guaranteeing the effectiveness of preventative measures [7]. Therefore, it is necessary to look again at the applicability of safe practices in all hospitals due to the scientific evidence for their effectiveness in reducing adverse events [8,9].

Within the scope of the mental health system, there is a shortage of studies on PSC, therefore, the analysis of the real problems related to safe practices in these environments is difficult, creating barriers both in perception and attitude related to safety in problematic areas, making effective planning and programming of interventions a challenge [10,11]. Patient safety culture can contribute to the successes of long-term treatments of the patients in mental health systems. Given this situation, a question arises: what is the perception of professionals working in the field of mental health regarding patient safety? The present study aimed to analyze the culture of patient safety, according to the perception of health professionals who work in the Psychosocial Care Network in the Western Amazon.

## 2. Methods

### 2.1. Study Design

A descriptive observational cross-sectional study with a quantitative approach was carried out with health professionals who work in the Psychosocial Care Network of the municipality of Rio Branco, state of Acre, located in the Brazilian Western Amazon.

The study population consisted of professionals from two health units, who provide direct assistance in mental health linked to the Brazilian Unified Health System, called SUS, and included: doctors, nurses, physiotherapists, technicians, and nursing assistants. Professionals in the administrative area were also included in the research for providing direct care to psychiatric patients. Data collection took place between January and April 2017, at the Acre Mental Health Hospital (HOSMAC) and at the Center for Psychosocial Care (CAPS AD III), for four weeks in each institution.

The study included professionals who had a minimum experience of six months in the service. All those who had no employment bond with the institutions where the questionnaire was applied (interns, academics, and residents) were excluded. The final sample resulted in 69 professionals who all responded to the questionnaire.

### 2.2. Measurements

To evaluate patient safety culture, a version of the questionnaire translated into Brazilian Portuguese: Hospital Survey On Patient Safety Culture (HSOPSC) was applied [7,12]. This instrument was created by the Agency for Healthcare Research and Quality (AHRQ) in the United States [13,14]. It is an instrument that, since 2004, has been in the public domain and has been widely used throughout the world to measure safety culture among hospital professionals, whose work influences patient therapy [15].

The instrument contains 50 items, of which 44 are related to specific issues of the safety culture and 6 to personal information. It includes: sociodemographic variables; variables of dimension of the safety culture within the unit (teamwork in the unit, expectations and actions to promote patient safety of the supervisor/manager, organizational learning and continuous improvement, feedback and communication regarding errors, openness to communications, staff and non-punitive responses to errors); dimension variables of the safety culture within the hospital organization (support from hospital management for patient safety, teamwork between hospital units, internal transfers and on-call shifts); outcome variables (general perception of patient safety, frequency of reported events); and two questions aimed at the global assessment of patient safety and the number of events reported by professionals in the last 12 months [16,17,18,19]. This instrument was translated into Brazilian Portuguese and was validated to meet the characteristics of Brazilian culture [7,16].

The study respected the ethical and legal aspects of research involving human beings, obtaining an opinion from the Research Ethics Committee (CEP) from the Federal University of Acre: Opinion 1,392,345, protocols no. 59/10 of 25 October 2010 and 11,113 of 29 November 2010.

### 2.3. Statistical Analysis

The HSOPSC data allowed statistical analyses to be carried out to verify whether the scores obtained in the dimensions of the hospital environment and/or level of the organization influenced the scores obtained in dimensions considered to be result variables [20]. The percentages classified as strong are the dimensions in which 75% of the subjects answered affirmatively to the questions asked in a positive way and negatively to the questions asked in a negative way. Critical areas were those that were in the 50–75% range of negative responses to positively formulated questions or positive responses to negatively formulated questions [13,14].

Each of the reliability criteria, regarding internal consistency, was examined by calculating the Alpha (α) Cronbach for items in the 12 dimensions of the questionnaire. Cronbach’s α is a measure of the internal consistency reliability of a measurement scale and assesses the extent to which items in a given dimension are interrelated. The minimum criterion for acceptable reliability is an α of at least 0.70. Reliability analyses verify the extent to which the measuring instrument, like a survey questionnaire, consistently measures the desired construction. Cronbach’s α ranges from 0 to 1, with higher values indicating greater reliability [21].

To organize the collected data, which were entered into an electronic spreadsheet in the Excel for Windows program, the percentage frequency of each dimension was calculated and classified, as recommended by AHRQ [13,14]. Regarding sociodemographic data, these were analyzed using descriptive statistics, with absolute counting and relative frequency for the data being performed. Reliability analysis was performed using the IBM SPSS version 22 program.

## 3. Results

The sample consisted of 69 professionals working at HOSMAC and CAPS AD III. Most health professionals work in the outpatient area (n = 42), equivalent to 60.9% of the answers. The most representative category was nursing technicians, which corresponded to 37.7% of respondents (Table 1).

In Table 1, information about the professionals’ length of service at the unit and in the labor sector is shown.

Regarding the service time of the professionals evaluated, it was observed that 31.9% of the interviewees were included in the period of 1 to 5 years of activity and 23.2% were included in the period of 6 to 10 years, showing high turnover in the area. The same pattern was observed with regard to the time that the professional worked in the same sector. It was observed that 31.9% of professionals were in the sector for 1 to 5 years, while 27.5% were in the same place for 6 to 10 years.

Concerning interaction with patients, it appeared that most professionals interacted and had direct contact (79.0%). Most professionals working in the units had a high weekly workload. Of the respondents, 44.9% worked 20 to 39 h per week, followed by 36.2%, who worked 40 to 59 h a week, which can be an indicator of overload.

Regarding the length of experience in their area of expertise, compared to the time they had been on the job, it was observed that most professionals were seniors. The vast majority of the data analyzed were in the group of 6 to 10 years (27.5%), followed by professionals with more than 21 years of professional experience (23.2%).

When evaluating the HOSMAC and CAPS AD III data, presented by HSOPSC, according to the professionals’ positive response rates (Table 2), it was observed that a lower level of education and knowledge on the topic led to a lower chance of reporting any adverse events. In this sense, the expectations and actions to promote safety by supervisors and managers presented the highest rate with 75%, followed by the support of hospital management for patient safety with 64%.

Cronbach’s α value was estimated for all items of the HSOPSC instrument. The global value was 0.86 and separately for all 12 dimensions, there was variability between 0.10 and 0.89. The dimensions: frequency of related events, open communication, and support from hospital management for patient safety presented the highest coefficients at 0.89, 0.79, and 0.79, respectively. However, the staffing and non-punitive responses to errors dimensions had values of 0.12 and 0.10, respectively, presenting the lowest coefficients (Table 3).

**Figure 1:**
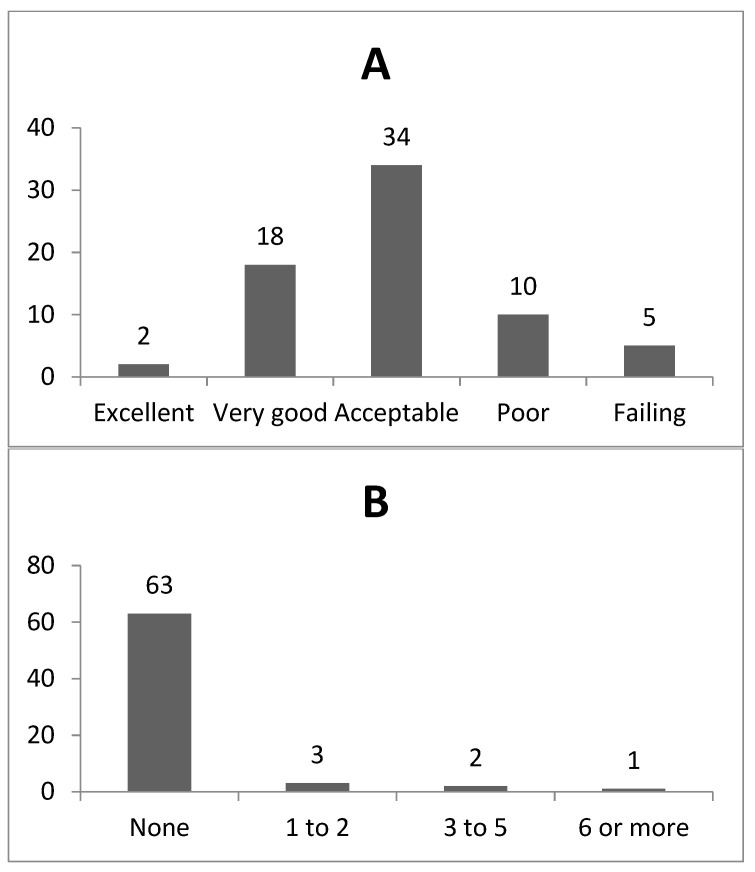
Absolute frequency of responses on the patient safety score (Panel **A**) and absolute frequency of responses on the number of adverse events reported in the last 12 months (Panel **B**) in the Psychosocial Care Network of the Municipality of Rio Branco, 2017 (n = 69).

Concerning the absolute frequency of responses on the patient safety score, 34 professionals from HOSMAC and CAPS AD III rated the PSC score as acceptable, 18 as very good, and 2 as excellent (Figure 1A). Regarding the number of adverse events reported in the two units evaluated, 63 professionals did not report any adverse events in the last 12 months (Figure 1B).

## 4. Discussion

The purpose of this work was to investigate the perception of the safety environment among professionals who are part of a multidisciplinary team working at the CAPS AD III and at the HOSMAC located in the Western Amazon of Brazil. The unit with the largest number of participating professionals was HOSMAC because it is the reference mental health hospital, while CAPS AD III is a general center for attending to people with mental health issues. In both institutions, health professionals generally have a close relationship with patients. However, it was observed that they had an extensive workload, comprising 40 to 59 h a week. This extensive workload leads to fatigue and stress, corroborating the occurrence of errors [22,23,24]. Professionals with this level of workload have a loss in performance of their routine activities and, consequently, have a less beneficial relationship with their professional performance [25,26]. One possible explanation for the observed turnover of professionals among sectors in both health units might be this workload. This could be associated with professional dissatisfaction and correlated with the occurrence of adverse events, such as medication errors, nosocomial infections, and falls [27,28]. Professionals who have worked at the same institution for years have more experience in the routine, positively impacting interpersonal relationships and resolving disagreements between team members [18,20], and also decreasing the probability doubts in relation to diagnostic and therapeutic approaches [26]. In addition, health professionals who worked for 21 years or more had a better perception of PSC [29], whereas other health professionals with 6 to up to 10 years of experience had a better perception of PSC than professionals with more time of experience [30].

It was evident in Table 2 that the domain “perception of hospital management” had high scores, showing alignment between hospital management and employees. This is an important factor in guaranteeing patient safety [31]. However, in relation to the “punitive responses to errors”, low scores were observed, indicating dissatisfaction and making it clear that the idea of pointing out errors generates punitive attitudes from the manager’s part. The punitive culture is still present within the health institution [31]. One way out of this situation is to create a multi-professional atmosphere in the work environment, providing an open dialogue about errors and non-punitive consequences aligned with continuous training of professionals [32]. Most errors cannot be prevented individually, but collectively, in order to avoid future errors on the part of the team as a whole. An improvement in communication between managers and professionals and among the employees themselves will address individual errors in a systemic way, decreasing fear of punishment [33]. Extensive literature shows that discussing and managing errors can facilitate the notification of incidents and the collective learning and knowledge of their root causes [19,21,24,34,35].

There is a need for improvements regarding the domain “organizational learning” and “feedback and communication” about errors. A Brazilian study identified a similar reality and inferred that professionals do not recognize working conditions as potential facilitators for the occurrence of errors, centering the responsibility for the quality and safety of work on professionals [32]. The need to strengthen a safety culture at the organizational level is pointed out as a fundamental measure in the process of improving patient safety in the hospital context [27]. For a positive safety culture, it is necessary that the institution’s managers emphasize safety, promote and encourage feedback, establish safety parameters, and enable the training of professionals [4,36]. In Brazil, studies indicate that the dissatisfaction of health professionals is related to the accumulation of activities and the scarce prospects of obtaining new knowledge, impairing the quality of their performance with the patient [9]. These weaknesses end up negatively impacting patient safety culture [37,38]. It is of utmost importance that all professionals involved in the management and operation of the evaluated units understand that the error is a great learning opportunity and not reprisals to colleagues in the face of situations that may put the patient at risk [37,39].

The data collection instrument is self-applicable, and some professionals who received it left certain items unmarked because they did not want to answer or because of the difficulties in understanding what was being questioned, which is considered as a limitation of this study. Another aspect refers to filling out the questionnaire during working hours, which was sometimes interrupted due the need of service, favoring the non-completion of responses by some participants. Lastly, a stratification analysis of the perception of PSC by professional role (physicians, nurses, and so on) could reveal other outcomes, given the assumption that professional role may influence this perception, according to the previous literature [40].

## 5. Conclusions

This study intended to corroborate the dynamics of expanding a database containing information on the process of evaluating safety culture over time, the impact that this evaluation can have on health units, possible interventions regarding research findings, and recognition of the organizational situation. In this way, managers in the mental health area can focus their actions on the observed weaknesses, improving the quality of care provided and patient safety globally, thus, promoting a culture of patient safety that will be an extremely tangible reality.

## Figures and Tables

**Table 1:** Distribution of the profile of health professionals according to HSOPSC variables in the Network of Psychosocial Care of the Municipality of Rio Branco, 2017.

Variables	N	%
**Area/Work Unit**		
Ambulatory of Mental Health	42	60.9
Emergency Sector	03	4.3
Pharmacy	03	4.3
Others	20	29.0
Total	69	100.0
**Office or function**		
Nursing technicians	26	37.7
Nurse	09	13.0
Physiotherapy	06	8.7
Psychologist	02	2.9
Pharmacist/Biochemist/Biomedical	01	1.4
Nutritionist	01	1.4
Other categories	06	8.7
No reply	18	26.1
Total	69	100.0
**Network Location**		
Acre Mental Health Hospital (HOSMAC)	62	89.9
Center for Psychosocial Care (CAPS AD III)	07	10.1
Total	69	100.0
**Interaction with patients**		
YES, I usually have interaction or direct contact with patients.	55	79.0
I DO NOT have interaction or direct contact with patients.	14	21.0
Total	69	100
**Time working in the hospital (Years)**		
Less than 1 year	06	8.7
1 to 5 years	22	31.9
6 to 10 years	16	23.2
11 to 15 years	05	7.2
16 to 20 years	05	7.2
21 years and over	15	21.7
Total	69	69
**Time working in the current area/unit of the hospital (Years)**		
Less than 1 year	04	5.8
1 to 5 years	22	31.9
6 to 10 years	19	27.5
11 to 15 years	05	7.2
16 to 20 years	11	15.9
21 years and over	08	11.6
Total	69	100.0
**Working Hours per Week (Hours)**		
Less than 20 h per week	01	1.4
20 to 39 h per week	31	44.9
40 to 59 h per week	25	36.2
60 to 79 h per week	09	13.0
No reply	03	4.3
Total	69	100.0
**Time working on your Current Specialty (Years)**		
Less than 1 year	03	4.3
1 to 5 years	14	20.3
6 to 10 years	19	27.5
11 to 15 years	09	13.0
16 to 20 years	07	10.1
21 years and over	16	23.2
No reply	01	1.4
Total	69	100.0

**Table 2:** Distribution of the positive response rate of health professionals according to the Dimensions of Patient Safety in the Network of Psychosocial Care of the Municipality of Rio Branco, 2017.

Patient Safety Dimensions Rate of Positive Responses (%)	Rate of Positive Responses (%)
Expectations and actions to promote the safety of supervisors and managers	75
Hospital management support for patient safety	64
Internal Transfers and Work Placement	56
Communication opening	52
Organizational learning	51
Teamwork within the units	49
Teamwork between units	49
Frequency of Reported Events	48
Feedback and Communication About Errors	43
Staffing	38
General Perceptions of Patient Safety	35
Non-punitive responses to errors	27

**Table 3:** Reliability Analysis (Cronbach’s α) of Patient Safety Dimensions, in the Psychosocial Care Network of the Municipality of Rio Branco, according to the Questionnaire on Hospital’s Patient Safety Culture (HSOPSC), 2017.

Factors (Items)	Cronbach’s Alpha	Silva-Batalha and Mellerio [22]	Reis et al. [16]	Tomazoni et al. [23]
All items from HSOPSC	0.86	0.90	-	-
Frequency of related events (D1, D2, D3)	0.89	0.87	0.91	0.88
Expectations and actions to promote the safety of supervisors and managers (B1, B2, B3R, B4R)	0.65	0.77	0.76	0.74
Feedback and communication about errors (C1, C3, C5)	0.77	0.67	0.72	0.72
Teamwork within the units (A1, A3, A4, A11)	0.68	0.68	0.66	0.61
Communication opening (C2, C4, C6R)	0.79	0.63	0.69	0.64
Hospital management support for patient safety (F1, F8, F9R)	0.79	0.72	0.84	0.60
Internal transfers and tickets on duty (F3R, F5R, F7R, F11R)	0.58	0.70	0.70	0.64
Non-punitive responses to errors (A8R, A12R, A16R)	0.10	0.40	0.35	0.47
Organizational learning (A6, A9, A13)	0.53	0.60	0.56	0.74
Teamwork between hospital units (F2R, F4, F6R, F10)	0.53	0.56	0.67	0.60
General safety perceptions (A10R, A15, A17R, A18R)	-	0.47	0.52	0.43
Staffing (A2, A5R, A7R, A14R)	0.12	0.66	0.20	0.46
